# Bio-Based Coating Materials Derived from Acetoacetylated Soybean Oil and Aromatic Dicarboxaldehydes

**DOI:** 10.3390/polym11111809

**Published:** 2019-11-04

**Authors:** Zhiyuan Cao, Fei Gao, Jinze Zhao, Xiao Wei, Qian Cheng, Jiang Zhong, Cong Lin, Jinbing Shu, Changqing Fu, Liang Shen

**Affiliations:** Jiangxi Engineering Laboratory of Waterborne Coating, School of Chemistry and Chemical Engineering, Jiangxi Science &Technology Normal University, Nanchang 330013, China; 13097208064@163.com (Z.C.); 15936691596@163.com (J.Z.); wxjxkjsfdx@163.com (X.W.); cqzylto@163.com (Q.C.); jiangzhong@jxstnu.com.cn (J.Z.); conglin0127@jxstnu.com.cn (C.L.); jinbingshu@jxstnu.com.cn (J.S.); fuchq0791@jxstnu.com.cn (C.F.)

**Keywords:** epoxidized soybean oil, acetoacetylated soybean oil, Knoevenagel condensation reaction, aromatic dicarboxaldehydes

## Abstract

Bio-based coating materials were prepared from epoxidized soybean oil as a renewable source. Acetoacetylated soybean oil was synthesized by the ring-opened and transesterification reaction of epoxidized soybean oil, and its chemical structure was characterized by nuclear magnetic resonance (NMR), Fourier transform infrared spectroscopy (FTIR), gel permeation chromatography (GPC), and rheometric viscosity analyses. On the basis of acetoacetylated soybean oil, several bio-based coating materials were prepared using different aromatic dicarboxaldehydes (1,2-benzenedialdehyde, 1,3-benzenedialdehyde, 1,4-phthalaldehyde, 4,4′-biphenyldicarboxaldehyde) and characterized. The resulting films possess good performance, including the highest glass transition temperature of 54 °C, a Young’s modulus of 24.91 MPa, tensile strength of 5.65 MPa, and an elongation at break of 286%. Thus, this work demonstrates the Knoevenagel condensation reaction, which is based on soybean oil as a potential newer eco-friendly raw material.

## 1. Introduction

Currently, the development of renewable resources to replace petroleum-based raw materials in the areas of energy and materials is a prominent research field [1,2,3]. Vegetable oil, as a renewable resource, has attracted considerable attention owing to its low cost, availability, low ecotoxicity, and environmental sustainability [4,5]. Vegetable oils contain unsaturated C–C bonds; thus, epoxidation of vegetable oils is commonly employed to graft functional groups by ring-opened reactions [6,7,8].

Acetoacetate chemistry is an important class of versatile synthetic intermediates of thermoset resins [9,10]. The functional groups of acetoacetates allow an efficient crosslinking reaction with various compounds such as isocyanates [11], acrylates (Michael Reaction) [12], amines (enamine formation) [13], and aldehydes. Although there are many synthetic routes for the preparation of covalently bonded acetoacetates into resins, only a few examples have been reported that are applicable for bio-based coating materials. In 2002, the Trumbo group reported a novel bio-based coating material, which is based on an acetoacetylated castor oil and multifunctional amines; the acetoacetylated castor oil was obtained by a transesterification reaction between castor oil and t-butyl acetoacetate, and the properties of the coating were improved by increasing the temperature [14]. In 2012, the Webster group developed a bio-based coating material by amine-acetoacetate reactions between acetoacetylated sucrose and multifunctional amines; the coatings exhibited a good balance of properties owing to the degree of enamine alkyl substitution or alkyl chain length [15]. Recently, our group reported a series of bio-based coating materials; the properties of these coatings were controlled by changing the content of acetoacetate groups by thiolene and transesterification reactions [16,17,18,19].

The Knoevenagel condensation reaction is as an important C–C bond forming reaction, which can be employed to synthesize many compounds such as herbicides [20], natural products [21], and functional polymers [22]. However, currently, few examples of bio-based coating materials have been reported, which utilized Knoevenagel condensation reactions. In 2019, our group reported the first bio-based coating materials, which were made via the Knoevenagel condensation reaction between acetoacetylated castor oil and aromatic dicarboxaldehydes; however, the coating had inferior mechanical properties [23].

**Scheme 1:**
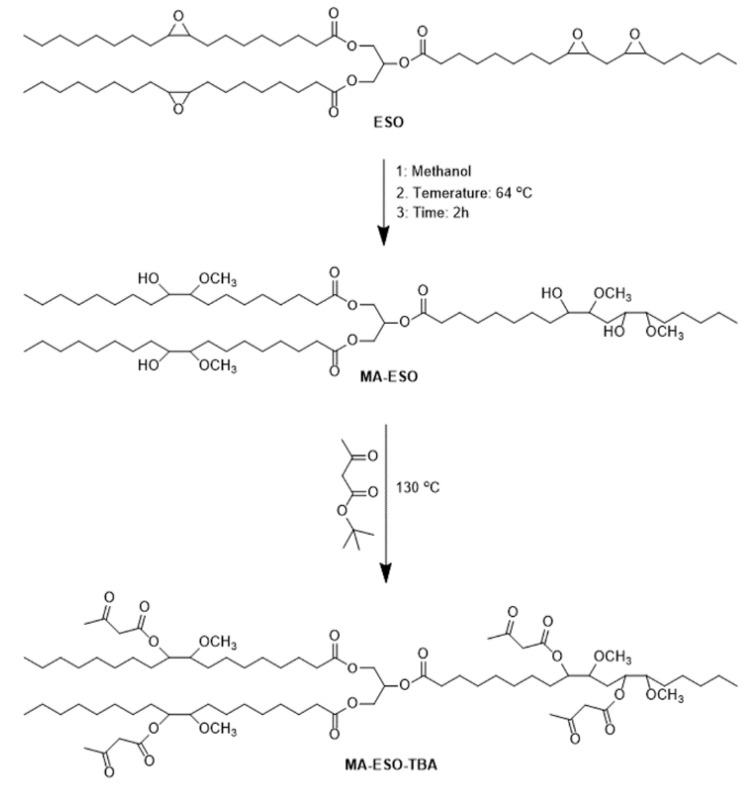
Synthesis of acetoacetylated soybean oil via the ring-opened and transesterification reactions [24].

In this paper, we show a novel bio-based coating material, which is based on the reaction between acetoacetylated soybean oil and aromatic dicarboxaldehydes. Acetoacetylated soybean oil was prepared by the ring-opened and transesterification reaction (Scheme 1) [24]. The primary novelties of this paper include the following: (1) Expanding a Knoevenagel condensation reaction base on acetoacetylated soybean oil, (2) obtaining good mechanical/chemical performance, and (3) demonstrating that this method has a very wide application value for renewable plant oils because vegetable oils contain many unsaturated C–C bonds and have easy access to epoxy structures.

## 2. Materials and Methods

### 2.1. Materials

Epoxidized soybean oil (ESO), 1,2-benzenedialdehyde, 1,4-phthalaldehyde, 1,3-benzenedialdehyde, t-butyl acetoacetate, tetrafluoro boric acid, and 4,4′-biphenyldicarboxaldehyde were obtained from Sigma Aldrich (Shanghai, China). Ethyl acetate, benzaldehyde, toluene, methanol, tetrahydrofuran (THF), and ethyl acetate were obtained from Xiya Reagent (Chengdu, Sichuang, China). 1,8-Diazabicyclo (5,4,0) undec-7-ene (DBU), 4-dimethylaminopyridine (DMAP), triethylamine, and piperidine were obtained from Beijing Chemical Works (Beijing, China). All materials were used without any further purification.

### 2.2. Preparation of Soybean Oil-Based Polyols

The synthesis of soybean oil-based polyols was performed according to a previously patent reported study [24] (Scheme 1). A mixture of epoxidized soybean oil (60 g, 62.5 mmol), methanol (80 g, 2.5 mol), and tetrafluoro boric acid (0.1 wt % of epoxidized soybean oil) was heated in a 250 mL flask at 64 °C with magnetic stirring for 2 h. The mixture was quenched to room temperature, extracted by ethyl acetate, and washed with a saturated sodium chloride solution three times. The organic solvent was removed via rotary evaporation, and a colorless oil was obtained with a yield of 84%. ^1^H NMR (CDCl_3_, 400 MHz): δ(ppm) = 5.22 (s, 1H), 4.28–4.24 (m, 2H), 4.13–4.12 (m, 2H), 3.99–3.73 (m, 4H), 3.62–3.56 (m, 2H), 3.37 (s, 12H), 3.33–3.25(m, 4H), 2.29–2.26 (m, 6H), 2.06–1.96 (m, 6H), 1.85–1.79 (m, 2H), 1.58–1.43 (m, 8H), 1.28–1.22 (m, 62H), and 0.85–0.82 (t, 12H). ^13^C NMR (CDCl_3_, 400 MHz): δ(ppm) = 173.14, 84.30, 82.68, 72.48, 68.92, 65.03, 62.04, 58.10, 56.94, 56.59, 42.7, 34.06, 33.37, 29.88, 29.59, 25.90, 25.05, 24.75, 23.71, 14.03, 13.92. GPC (theoretical formula weight = 1089); *M*_n_ = 1006, *M*_w_ = 1057, PDI = 1.05. Viscosity: 1.31 Pa s.

### 2.3. Preparation of Acetoacetylated Soybean Oil

The synthesis of acetoacetylated soybean oil was performed according to a previously patent reported study [24] (Scheme 1). A solution of soybean oil-based polyols (63 g, 57.9 mmol) and t-butyl acetoacetate (36.64 g, 0.232 mol) in a 250 mL reflux condenser flask was prepared, and then the reaction was heated with magnetic stirring to 110 °C under nitrogen atmosphere. At 100–110 °C, the liquid of n-butanol was removed, and a yellow oil was obtained with a yield of 85%. ^1^H NMR (CDCl_3_, 400 MHz): δ(ppm) = 5.21–5.16 (m, 1H),4.96–4.92 (m, 4H), 4.23–4.20 (m, 2H), 4.09–4.07 (m, 2H), 3.41 (s, 4H), 3.32 (s, 12H), 2.27–2.23 (t, 6H), 2.19 (s, 12H), 2.27–2.23 (m, 6H), 1.55–1.49 (m, 6H), 1.58–1.43 (m, 8 H), 1.22–1.18 (m, 62 H), 0.81–0.78 (m,12H). ^13^C NMR (CDCl_3_, 400 MHz): δ(ppm) = 199.20, 172.09, 165.76, 80.23, 74.14, 67.78, 60.90, 57.28, 55.78, 49.07, 32.84, 30.76, 28.54, 27.97, 24.39,23.66, 21.44, 12.94. GPC (theoretical formula weight = 1426); *M*_n_ = 1169, *M*_w_ =1228, PDI = 1.05. Viscosity: 0.61 Pa s.

### 2.4. Polymerization of Bio-Based Coating Materials

**Scheme 2:**
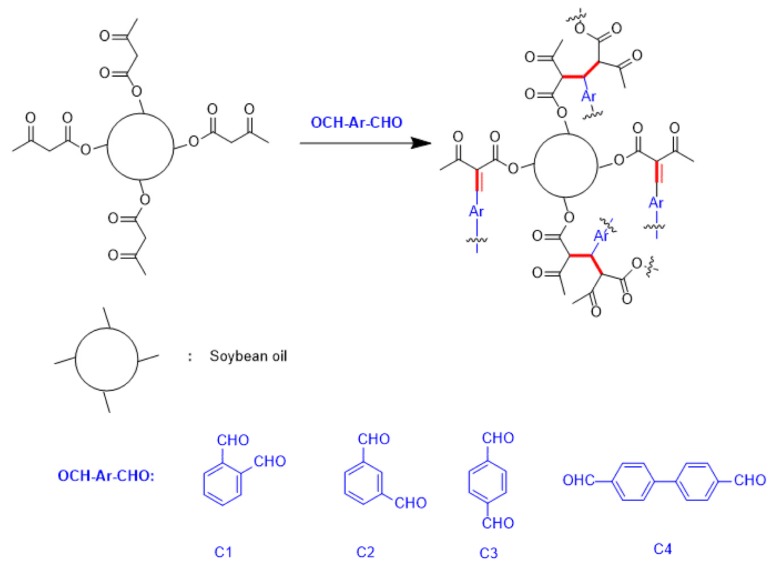
Synthetic route for coating materials.

The method used for the preparation of the coating materials is as follows (Scheme 2 and Table 1). A mixture of acetoacetylated soybean oil (1 g, 0.7 mmol) and crosslinker agent (1,2-benzenedialdehyde, 1,3-benzenedialdehyde, 1,4-phthalaldehyde, 4,4′-biphenyldicarboxaldehyde) (1.05 mmol) was dissolved in THF (3 mL) for 10 min (owing to the incompatibility of aromatic dicarboxaldehydes with acetoacetylated soybean oil, the coating of materials was performed in a solvent). Then, the catalyst (5 wt % of acetoacetylated soybean oil, DBU) was added to the mixture and mixed for another 10 min. Then, the mixture solution was poured into a poly(tetrafluoroethylene) (PTFE) mould (8 cm × 8 cm × 1.5 cm) and cured at ambient temperature (25 °C) to obtain a dry coating film (200–300 μm thick). The complete curing reaction was determined by FTIR.

### 2.5. Characterization

^1^H and ^13^C NMR spectra were obtained using a Bruker AV-400 NMR (Bruker, Rheinstetten, Germany) with tetramethylsilane as an internal reference, and deuterated chloroform (CDCl_3_) was used as a solvent. Infrared spectra (IR) were obtained using a Bruker-Vertex70 spectrometer (Bruker, Rheinstetten, Germany) in the attenuated total reflection (ATR) mode. An average of 32 scans of each sample in the range from 3500 cm^−1^ to 500 cm^−1^ was obtained. The viscosities of different components and monomer mixtures were measured using a rheometer (Discovery HR-2 rheometer) (TA Instruments, New Castle, DE, USA) at room temperature (25 °C). The drying times for the coatings were determined using a BK Drying Recorder (MICKLE Laboratory Engineering Co. Ltd., Gomshall, United Kingdom), and the results were analyzed using an ASTM D5895–2013. Thermogravimetric analysis (TGA) was performed using a TGA Q50 system (TA Instruments) (TA Instruments Q800, New Castle, DE, USA) at a heating rate of 10 °C min^−1^ under an N_2_ atmosphere flow of 80 mL/min. Tensile property was determined using a dynamic mechanical analyzer (DMA) (TA Instruments Q800, New Castle, DE, USA) with a cross-headspeed of 10 mm/min at 25 °C. Rectangular films of 30 mm × 3 mm (length × width) were used for the test, for the final tensile strength and elongation at break of all the films were obtained from average values of at least three replicates of each sample. A dynamic mechanical analyzer (DMA) (TA Instruments Q800, New Castle, DE, USA) in tensile mode at 1 Hz was used to study the storage moduli and tan *δ* under controlled temperature. The samples were heated from −30 °C to 110 °C at a rate of 5 °C min^−1^. Gel permeation chromatography (GPC) was performed on a GPC apparatus (Waters 515; Waters, MA, USA) at 25 °C and a 5 μm Styragel HR5E column (Waters, MA, USA). Samples were diluted to 2 mg mL^−1^ in tetrahydrofuran (THF) for GPC analysis. THF was used as an eluent at a flow rate of 1.0 mL min^−1^. Molecular weights were determined using polystyrene standards (The system was calibrated by narrow polystyrene standards (*M*_w_ range: 200–300,000 g/mol)). The gel content was determined by fully immersing a known mass (*W*_1_) of cured material in tetrahydrofuran for 24 h at ambient temperature, followed by filtering, drying, and reweighing of the solid remnants W_2_. The gel content was calculated by the following equation: *M* (%) = *W*_2_/*W*_1_ × 100%. The tetrahydrofuran (THF) swelling of film was performed by immersing a known weight (*W*_0_) of the film in a tetrahydrofuran bath, and the towel-dried sample weight (*W*_t_) was obtained. The swelling of the film was calculated by the following equation: Q (%) = [(*W*_t_ − *W*_0_)/*W*_0_] × 100%.

## 3. Results and Discussion

### 3.1. Characterization of AcetoacetylatedSoybean Oil

**Figure 1:**
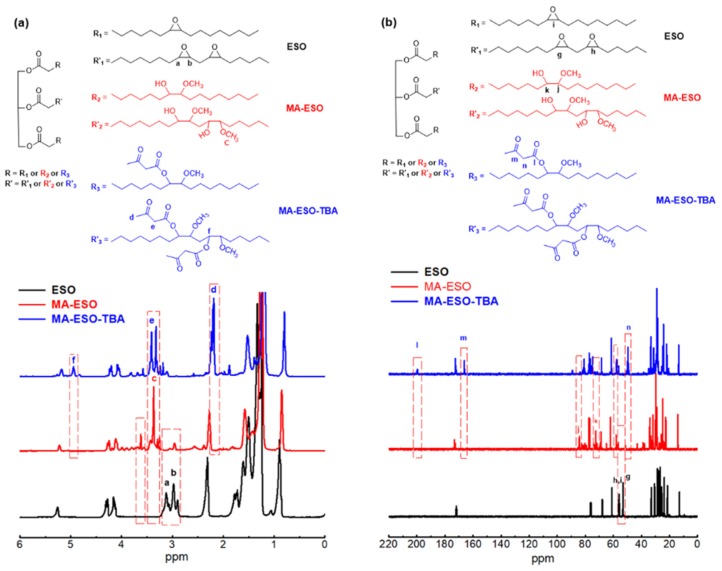
NMR spectra of acetoacetylated soybean oil: (**a**) ^1^H NMR spectra and (**b**) ^13^C NMR spectra.

In this study, the oxirane group number of ESO used was 4 per molecule, which was confirmed by ^1^H NMR spectroscopy (Figure S1a, Supporting Information). The chemical structure of the obtained acetoacetylated soybean oil was confirmed by nuclear magnetic resonance spectroscopy (NMR), GPC analyses, and Fourier transform infrared spectroscopy (FTIR). As shown in Figure 1a, the peaks (soybean oil-based polyols, red) at 2.9 ppm (b) and 3.1 ppm (a), which correspond to the oxirane group of epoxidized soybean oil (black), decreased after the ring-opened reaction (by approximately 84%, Figure S1b, Supporting Information), and a new peak (soybean oil-based polyols, red) at 3.6 ppm (c) appeared, which is assigned to the methyl of methanol grafted to epoxidized soybean oil (red). By comparing soybean oil-based polyols (red) to acetoacetylated soybean oil (blue), it was determined that the peaks (soybean oil-based polyols, red) at 3.9 ppm and 4.0 ppm shifted to 4.8–4.9 ppm (acetoacetylated soybean oil), and the peaks corresponding to the new acetoacetate groups appeared at 3.4 and 2.2 ppm (approximately 85%, in Figure S2b, Supporting Information). In addition, the ^13^C NMR spectra confirmed that acetoacetylated soybean oil was obtained. The peaks (soybean oil-based polyols, red) attributed to the epoxy bonds in soybean oil (black) at 55 ppm (g) and 56 ppm (h) decreased after the ring-opened reaction, and the new peaks of acetoacetylated soybean oil (blue) at 200 ppm (l) and 165 ppm (m) are shown in Figure 1b. Comparing the gel permeation chromatography (GPC) curves of ESO, soybean oil-based polyols (MA-ESO), and acetoacetylated soybean oil (MA-ESO-TBA) (Figure S3, Supporting Information), the average molecular weight was increased by ring-opened and transesterification reactions.

**Figure 2:**
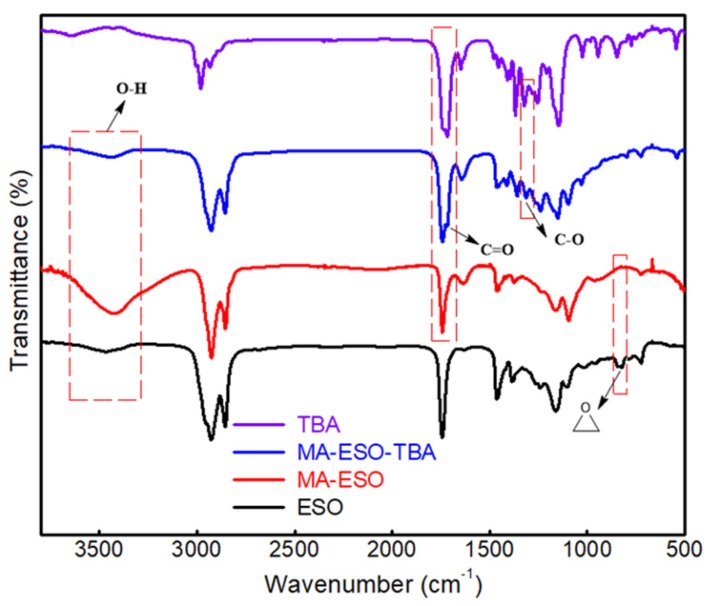
FTIR characterization of acetoacetylated soybean oil.

The FTIR spectra of ESO, soybean oil-based polyols (MA-ESO), acetoacetylated soybean oil (MA-ESO-TBA), and t-butyl acetoacetate are shown in Figure 2. The comparison of ESO (black) and MA-ESO (red) shows that the peak at 823 cm^−1^ (corresponded to epoxy groups) disappeared and a new peak at 3600–3300 cm^−1^ (OH group) appeared, which indicates that the ring-opened reaction of epoxidized soybean oil occurred to obtain hydroxyl groups. By comparing MA-ESO (red) and MA-ESO-TBA (blue), the peaks at 1731–1712 cm^−1^ (the stretching vibration band of C=O of–COCH_2_COCH_3_) and 1350 cm^−1^ (C–O stretch from the ester bond of–COCH_2_COCH_3_) were observed. These results indicated that acetoacetylated soybean oil was obtained by the ring-opened and transesterification reaction.

**Figure 3:**
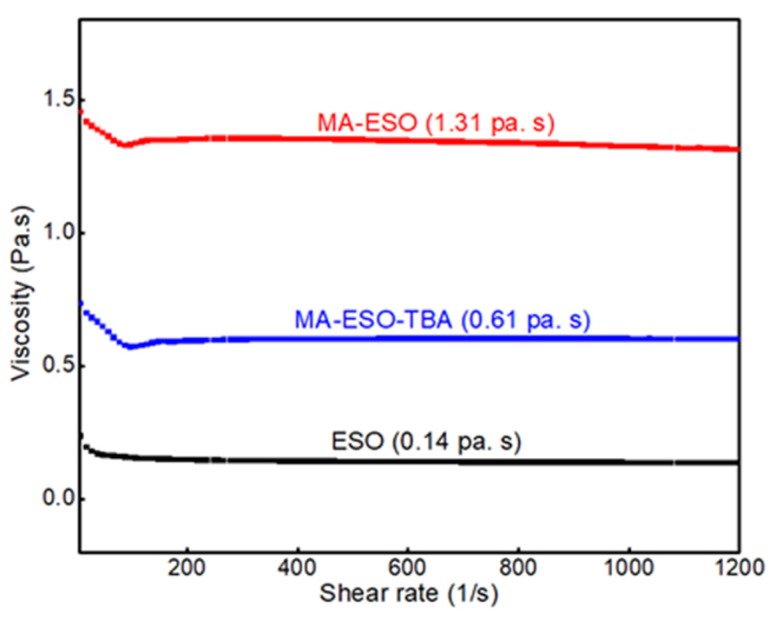
Rheological viscosity versus shear rate for epoxidized soybean oil (ESO), soybean oil-based polyols (MA-ESO), and acetoacetylated soybean oil (MA-ESO-TBA).

The viscosities of ESO, soybean oil-based polyols (MA-ESO), and acetoacetylated soybean oil (MA-ESO-TBA) were also measured. As shown in Figure 3, the viscosity increased from 0.14 Pa s (epoxidized soybean oil, black) to 1.31 Pa s (soybean oil-based polyols, red) after the ring-opened reaction.The main reason for thisis the increased intermolecular hydrogen bonding by the ring-opened reaction.Then, after the transesterification reaction (from soybean oil-based polyols to acetoacetylated soybean oil), the viscosity decreased from 1.31 Pa s to 0.61 Pa s, due to the disappeared hydrogen bonding interactions by the transesterification reaction [25]. However, compared to ESO and MA-ESO-TBA, the viscosity of MA-ESO-TBA was increased.The main reason for this may be that the molecular weight of MA-ESO-TBA wasincreased [26].

### 3.2. Characterization of the Films

In the initial study, we chose acetoacetylated soybean oil (MA-ESO-TBA) and 1,2-benzenedialdehyde as model starting materials to examine these coating materials, and the ratio of acetoacetate and aldehyde groups was 1:0.75, as previously reported [23]. As shown in Table 2, the film can be cured with piperidine as a catalyst (3 wt %) in 40 h (Table 2, Entry 1) and the curing time with DMAP (3 wt %) as a catalyst was 20 h (Table 2, Entry 2). When 1,8-diazabicyclo (5,4,0) undec-7-ene (DBU) (3 wt %) was used as acatalyst, the curing time could be reduced to 8 h with a good gel content of 83% (Table 2, Entry 3). Furthermore, we optimized the catalyst loading (DBU) and found that 5 wt % of DBU achieved the best curing time of 3.5 h, with a gel content of up to 88%.

In order to explanate the Knoevenagel reaction mechanism of this cured film, we used ethyl acetate and benzaldehyde as model materials, 5 wt % of DBU as catalyst (similar reaction condition based on the best optimize reaction condition), and found that two types of compounds were obtained and determined by NMR (Model reaction section, Supporting Information). The result showed that the acetoacetyloxy group can react with the aldehyde group and obtained two structure types at molar ratios of 1:0.75. Similar Knoevenagel reaction mechanisms have been reported in the Raju group [27].

After the optimization of reaction conditions, four coating (P1, P2, P3, and P4) films were obtained by different crosslinkers (C1, C2, C3 and C4, respectively). The characterization data of four coating films are displayed in Table 3. Comparison of the four films shows that all films had good gel content (87–90%). Thesolvent-swelling (in THF) decreasedfrom 220% to 130%; the main reason for this was that the crosslinking density from P1 to P4 is gradually increased.

#### 3.2.1. FTIR Characterization of Films

**Figure 4:**
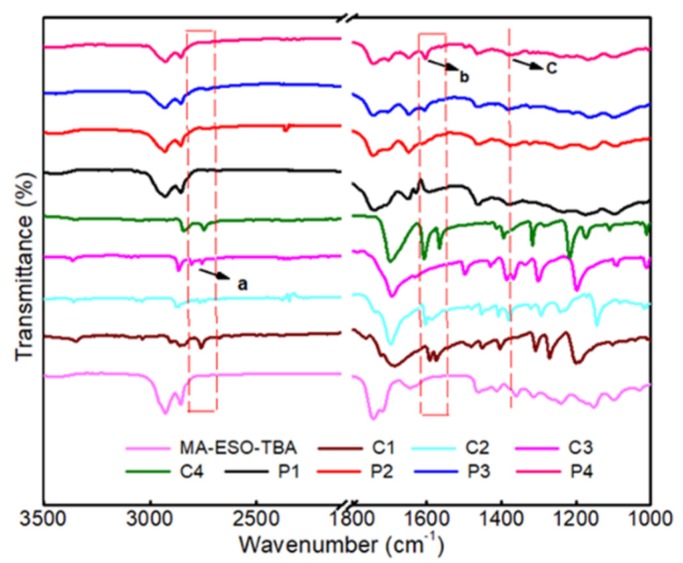
FTIR spectra of the four films (P1, P2, P3, and P4), four crosslinkers (C1, C2, C3, and C4) and acetoacetylated soybean oil, and the peaks of a is CHO stretching frequency, b and c are the peaks of benzene rings.

These coating films were characterized by FTIR, and the spectra are shown in Figure 4. Acetoacetylated soybean oil spectrum was compared with those of the samples C1 and P1, C2 and P2, C3 and P3, and C4 and P4, which were produced by the Knoevenagel addition reaction. The peaks for P1, P2, P3, and P4 at 2745 cm^−1^ (a, –CHO stretching frequency) decreased after curing, and the gels of the coating materials (P1, P2, P3, and P4) had characteristic absorption peaks of benzene rings at 1600 cm^−1^ (b) and 1381 cm^−1^ (c). These results indicated that four coating films were successfully obtained. Similar reactions between aldehyde and acetoacetyloxy groups have been previously reported [23,27,28].

#### 3.2.2. Mechanical Properties

**Figure 5:**
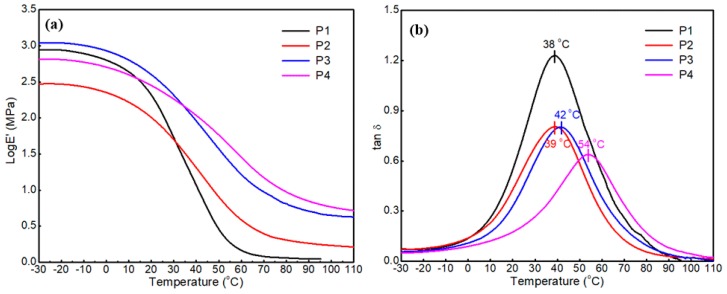
Dynamic mechanical analysis (DMA) of the four films: (**a**) Storage modulus and (**b**) loss factor (tan δ) as a function of temperature of the four films.

Figure 5 shows the storage modulus and loss factor (tan *δ*) as a function of temperature for the four films with different crosslinkers (C1, C2, C3, and C4). It was observed that all films exhibited similar trends of the storage modulus (E’) values, as follows: Storage modulus decreased at a certain temperature and then stabilized (Figure 5a). The loss factors (tan *δ*) of the four films are shown in Figure 5b. Specifically, only one peak is observed, which indicates that the four films exhibit homogeneous properties. The crosslinking density can be calculated by the equation *V*_e_ = E’/3RT, which has been previously reported [29,30,31]. The crosslinking density, *T*_g_, and storage modulus of the four films (P1–P4) are shown in Table 4. Compared to the four films, P4 has the higher *T*_g_ and crosslinking density. The main reason for this may be that the crosslinker C4 has alower steric hindrance structure.

**Figure 6:**
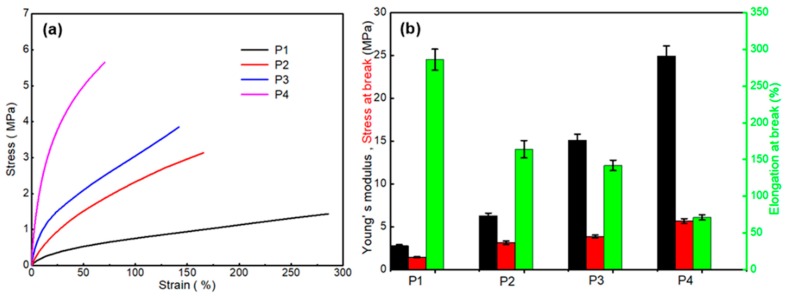
Stress-strain curves for the films, (**a**) DMA curves of these films (**b**) Young’s Modulus, stress at Break, and tensile strength of these cured samples.

The stress-strain behaviours of the four films are shown in Figure 6. By comparing P1, P2, and P3, it was determined that as the structural steric resistance of the crosslinkers (C1, C2, and C3, respectively) decreased, the crosslinking density and mechanical properties (elongation at break, Young’s modulus, and tensile strength) gradually increased (Table 4). Furthermore, P4 has the highest mechanical properties (elongation at break, Young’s modulus, and tensile strength), due to that P4 has the highest crosslinking density.

#### 3.2.3. Thermal Stability

**Figure 7:**
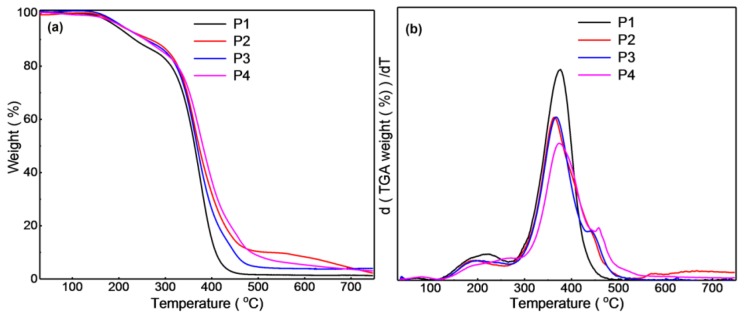
Curves (**a**) and DTGA curves (**b**) for the four films.

Figure 7 shows TGA curves and their derivative curves for the four films, and the *T*_10_, *T*_50_, and *T*_max_ data are summarized in Table 5. The thermal degradation profile of the four films shows two rapid thermal degradation steps at approximately 160–280 °C and 280–550 °C. The unstable ester groups were released at approximately 160–280 °C [32], and the chain scission of the corresponding polymer skeleton occurred at approximately 280–550 °C [33]. By comparing the *T*_10_, *T*_50_, and *T_max_* data of the four films, it was determined that the thermal stability of these films follows the order of P4 > P3 > P2 > P1. The glass transition temperature (*T*_g_) values determined from the differential scanning calorimetry (DSC) thermograms are shown in Figure S4 (in Supporting Information) and Table 5. Three films (P1, P2, and P3) have similar *T_g_*, and P4 has a higher *T_g_*. These results are similar to the glass transition temperature (*T*_g_) determined by DMA analysis.

*T*_10_, *T*_50_, and *T*_max_ represent the temperatures that correspond to the mass loss of 10 wt %, 50 wt %, and the maximum mass loss, respectively.

## 4. Conclusions

A novel bio-based coating material was developed using the Knoevenagel condensation reaction between acetoacetylated soybean oil and aromatic dicarboxaldehydes. Acetoacetylated soybean oilwas synthesizedat ambient curing conditions, and its chemical structure was determined by NMR, FTIR, GPC, and viscosity characterization. Then, four bio-based coating materials were prepared by acetoacetylated soybean oil and aromatic dicarboxaldehydes. These coating materials were characterized by Fourier transform infrared spectroscopy. Thermal and mechanical properties were characterized by DSC, TGA, and DMA. These coating materials exhibit good mechanical/chemical performance. The characteristics of this novel system demonstrate theapplicability of theKnoevenagel condensation reaction for soybean oil as a new eco-friendly raw material.

## Figures and Tables

**Table 1:** Sample codes and ratios of materials in the Knoevenagel addition reaction.

Sample Code	Acetoacetylated Soybean Oil	Crosslinker	Acetoacetate: Aldehyde Ratio
P1	1 g (0.7 mmol)	1,2-benzenedialdehyde (C1) (0.141 g, 1.05 mmol)	1:0.75
P2	1 g (0.7 mmol)	1,3-benzenedialdehyde (C2) (0.141 g, 1.05 mmol)	1:0.75
P3	1 g (0.7 mmol)	1,4-phthalaldehyde (C3) (0.141 g, 1.05 mmol)	1:0.75
P4	1 g (0.7 mmol)	4,4′-biphenyldicarboxaldehyde (C4) (0.221 g, 1.05 mmol)	1:0.75

**Table 2:** Optimization of the preparation reaction conditions.

Entry	Catalyst	CuringTime ^a^ (h)	Solvent-Swelling (%)	Gel Content (%)
1	Piperidine (3 wt %)	40 h	315	70
2	DMAP (3 wt %)	20 h	302	72
4	DBU (3 wt %)	8 h	270	83
5	DBU (5 wt %)	3 h	220	88
6	DBU (8 wt %)	2.8 h	225	85

^a^ Dry through curing time.

**Table 3:** Films made with different crosslinkers.

Sample	Curing Time (h)				Solvent-Swelling (%)	Gel Content (%)
	Set to Touch	Tack Free	Dry Hard	Dry Through		
P1	1.1	2.1	2.6	3 h	220	88
P2	1.2	3.2	4.6	5.5 h	175	90
P3	1.5	3.3	5.0	6 h	156	92
P4	1.6	4.1	5.6	6.5 h	130	87

**Table 4:** Mechanical properties of the films.

Sample Code	Tan δ	*T*_g_ (tan δ)	E’ at *T*_g_ + 50 °C (MPa)	Crosslinking (*V*e) (mol/m^3^)	Young’s Modulus (MPa)	Stress at Break (MPa)	Elongation at Break
P1	1.22	38 °C	0.13	14.34	2.76 ± 0.7	1.44 ± 0.05	286 ± 8
P2	0.81	39 °C	0.32	35.46	6.27 ± 1.6	3.54 ± 0.07	164 ± 6
P3	0.79	42 °C	0.79	88.82	15.07 ± 2.7	3.85 ± 0.1	142 ± 3
P4	0.63	54 °C	0.85	90.44	24.91 ± 3.9	5.65 ± 0.13	71 ± 1

**Table 5:** Thermal properties of the four films.

Sample	TGA in Nitrogen (°C)			DSC *T*_g_ (°C)
	*T*_10_	*T*_50_	*T*_max_	
P1	234	363	449	16
P2	268	372	473	14
P3	257	368	485	17
P4	256	381	531	34

## Supplementary Materials

The following are available online at https://www.mdpi.com/2073-4360/11/11/1809/s1, Figure S1 ^1^H NMR spectra of (a) epoxy soybean oil (ESO) and (b) soybean oil-based polyols (MA-ESO), Figure S2 ^1^H NMR spectra of (a) soybean oil-based polyols (MA-ESO) and (b) acetoacetylated soybean oil (MA-ESO-TBA). Figure S3 The GPC of acetoacetylated soybean oil, Figure S4 DSC curves indicate the glass transition temperature (*T*_g_) of the four films. Figure S5 ^1^H NMR (400 M) spectrum of epoxy soybean oil (ESO), Figure S6 ^13^C NMR (400 M) spectrum of epoxy soybean oil (ESO),Figure S7 ^1^H NMR (400 M) spectrum of soybean oil-based polyols (MA-ESO), Figure S8 ^13^C NMR (400 M) spectrum of soybean oil-based polyols (MA-ESO), Figure S9 ^1^H NMR (400 M) spectrum of acetoacetylated soybean oil (MA-ESO-TBA), Figure S10 ^13^C NMR (400 M) spectrum of acetoacetylated soybean oil (MA-ESO-TBA), Figure S111H NMR (400 M) spectrum of compound A, Figure S12 ^13^C NMR (400 M) spectrum of compound A.Table S1. Properties of the acetoacetylated soybean oil.

## References

[1] Dai J., Peng Y., Teng N., Liu Y., Liu C., Shen X., Mahmud S., Zhu J., Liu X. (2018). High-Performing and Fire-Resistant Biobased Epoxy Resin from Renewable Sources. ACS Sustain. Chem. Eng..

[2] Yao K., Tang C. (2013). Controlled Polymerization of Next-Generation Renewable Monomers and Beyond. Macromolecules.

[3] Allauddin S., Narayan R., Raju K.V.S.N. (2013). Synthesis and Properties of Alkoxysilane Castor Oil and Their Polyurethane/Urea–Silica Hybrid Coating Films. ACS Sustain. Chem. Eng..

[4] Chen R.Q., Zhang C.Q., Kessler M.R. (2015). Polyols and Polyurethanes Prepared from Epoxidized Soybean Oil Ring-Opened by Polyhydroxy Fatty Acids with Varying OH Numbers. J. Appl. Polym. Sci..

[5] Tsujimoto T., Takayama T., Uyama H. (2015). Biodegradable Shape Memory Polymeric Material from Epoxidized Soybean Oil and Polycaprolactone. Polymers.

[6] Biswas A., Sharma B.K., Willett J.L., Advaryu A., Erhan S.Z., Cheng H.N. (2008). Azide Derivatives of Soybean Oil and Fatty Esters. J. Agric. Food Chem..

[7] Tan J., Liu B., Fu Q., Wang L., Xin J., Zhu X. (2019). Role of the Oxethyl Unit in the Structure of Vegetable Oil-Based Plasticizer for PVC: An Efficient Strategy to Enhance Compatibility and Plasticization. Polymers.

[8] Dworakowska S., Bogdal D., Prociak A. (2012). Microwave-Assisted Synthesis of Polyols from Rapeseed Oil and Properties of Flexible Polyurethane Foams. Polymers.

[9] Wright T., Tomkovic T., Hatzikiriakos S.G., Wolf M.O. (2019). Photoactivated Healable Vitrimeric Copolymers. Macromolecules.

[10] Sims M.B., Lessard J.J., Bai L., Sumerlin B.S. (2018). Functional Diversification of Polymethacrylates by Dynamic β-Ketoester Modification. Macromolecules.

[11] Liu Z., Yu C., Zhang C., Shi Z., Yin J. (2019). Revisiting Acetoacetyl Chemistry to Build Malleable Cross-Linked Polymer Networks via Transamidation. ACS Macro Lett..

[12] Whittington C.P., Daily L.A., Miller K.M. (2014). Crosslinked imidazolium-containing polyester networks containing a pendant imidazolium group: Swelling studies and thermal properties. Polymer.

[13] Denissen W., De Baere I., Van Paepegem W., Leibler L., Winne J., Du Prez F.E. (2018). Vinylogous Urea Vitrimers and Their Application in Fiber Reinforced Composites. Macromolecules.

[14] Trevino A.S., Trumbo D.L. (2002). Acetoacetylated castor oil in coatings applications. Prog. Org. Coat..

[15] Xiao P., Nelson T.J., Webster D.C. (2012). Novel biobased dual-cure coating system. Prog. Org. Coat..

[16] Xu D., Cao Z., Wang T., Zhao J., Zhong J., Xiong P., Wang J., Gao F., Shen L. (2019). Effect of the Ratio of Acetylacetate Groups on the Properties of a Novel Plant-Based Dual-Cure Coating System. ACS Omega.

[17] Wang T., Wang J., He X., Cao Z., Xu D., Gao F., Zhong J., Shen L. (2019). An Ambient Curable Coating Material Based on the Michael Addition Reaction of Acetoacetylated Castor Oil and Multifunctional Acrylate. Coatings.

[18] Zuo H., Cao Z., Shu J., Xu D., Zhong J., Zhao J., Wang T., Chen Y., Gao F., Shen L. (2019). Effect of structure on the properties of ambient-cured coating films prepared via a Michael addition reaction based on an acetoacetate-modified castor oil prepared by thiol-ene coupling. Prog. Org. Coat..

[19] Xu D., Cao Z., Wang T., Zhong J., Zhao J., Gao F., Luo X., Fang Z., Cao J., Xu S. (2019). An ambient-cured coating film obtained via a Knoevenagel and Michael addition reactions based on modified acetoacetylated castor oil prepared by a thiol-ene coupling reaction. Prog. Org. Coat..

[20] Kastl J., Braun J., Prestel A., Möller H.M., Huhn T., Mayer T.U. (2015). Mad2 Inhibitor-1 (M2I-1): A Small Molecule Protein–Protein Interaction Inhibitor Targeting the Mitotic Spindle Assembly Checkpoint. ACS Chem. Biol..

[21] Peña R., Martín P., Feresin G.E., Tapia A., Machín F., Estévez-Braun A. (2016). Domino Synthesis of Embelin Derivatives with Antibacterial Activity. J. Nat. Prod..

[22] Bi S., Lan Z.A., Paasch S., Zhang W., He Y., Zhang C., Liu F., Wu D., Zhuang X., Brunner E. (2017). Substantial Cyano-Substituted Fully sp2-Carbon-Linked Framework: Metal-Free Approach and Visible-Light-Driven Hydrogen Evolution. Adv. Funct. Mater..

[23] He X., Zhong J., Cao Z., Wang J., Gao F., Xu D., Shen L. (2019). An exploration of the Knoevenagel condensation to create ambient curable coating materials based on acetoacetylated castor oil. Prog. Org. Coat..

[24] Campbell C.J., Lewandowski K.M., Owusu-Adom K. (2016). Curable and Cured Compositions. U.S. Patent.

[25] Yin Y., Ma L., Wen S., Luo J. (2018). Fracture of the Intermolecular Hydrogen Bond Network Structure of Glycerol Modified by Carbon Nanotubes. J. Phys. Chem. C.

[26] Yadav S.K., Hu F., La Scala J.J., Palmese G.R. (2018). Toughening Anhydride-Cured Epoxy Resins Using Fatty Alkyl-Anhydride-Grafted Epoxidized Soybean Oil. ACS Omega.

[27] Allauddin S., Jena K.K., Mishra A.K., Radhika K.R., Narayan R., Raju K.V.S.N. (2012). Synthesis & characterization of benzaldehyde modified acetoacetylated polyesters for polyurethane/urea coatings. Prog. Org. Coat..

[28] Dalessandro E.V., Collin H.P., Guimarães L.G.L., Valle M.S., Pliego J.R. (2017). Mechanism of the Piperidine-Catalyzed Knoevenagel Condensation Reaction in Methanol: The Role of Iminium and Enolate Ions. J. Phys. Chem. B.

[29] Mehta B., Watt P., Soucek M.D., Pugh C. (2016). Moderate Temperature Curing of Plant Oils with Bismaleimides via the Ene Reaction. Ind. Eng. Chem. Res..

[30] Zhang Y., Li Y., Guan Q., Liang G., Gu A. (2017). Developing self-healable and antibacterial polyacrylate coatings with high mechanical strength through crosslinking by multi-amine hyperbranched polysiloxane via dynamic vinylogous urethane. J. Mater. Chem. A.

[31] Zhang C., Madbouly S.A., Kessler M.R. (2015). Biobased Polyurethanes Prepared from Different Vegetable Oils. ACS Appl. Mater. Interfaces.

[32] Fu C., Liu J., Xia H., Shen L. (2015). Effect of structure on the properties of polyurethanes based on aromatic cardanol-based polyols prepared by thiol-ene coupling. Prog. Org. Coat..

[33] Feng Y., Liang H., Yang Z., Yuan T., Luo Y., Li P., Yang Z., Zhang C. (2017). A Solvent-Free and Scalable Method to Prepare Soybean-Oil-Based Polyols by Thiol–Ene Photo-Click Reaction and Biobased Polyurethanes Therefrom. ACS Sustain. Chem. Eng..

