# Supplementary material for: Bio-Based Coating Materials Derived from Acetoacetylated Soybean Oil and Aromatic Dicarboxaldehydes
Source: Polymers (Basel). 2019 Nov 4;11(11):1809. doi: 10.3390/polym11111809 (PMC6918255; doi:10.3390/polym11111809)
Supplement: Supplementary file 1 [file polymers-11-01809-s001.pdf]

polymers

MDPI

Supporting Information For

Bio-Based Coating Materials Derived from Acetoacetylated Soybean Oil and Aromatic Dicarboxaldehydes

Zhiyuan Cao, Fei Gao *, Jinze Zhao, Xiao Wei, Qian Cheng, Jiang Zhong, Cong Lin, Jinbing Shu, Changqing Fu, Liang Shen *

Jiangxi Engineering Laboratory of Waterborne Coating, School of Chemistry and Chemical Engineering, Jiangxi Science &Technology Normal University, Nanchang 330013, Jiangxi, P. R. China

E-mail address: feigao2016@jxstnu.com.cn (F. Gao);liangshen@jxstnu.com.cn (L. Shen).

Table of Contents

1. Calculation of the yield of acetoacetylated soybean oil by ¹H NMR 1

1.1 Calculation of the yield of soybean oil-based polyols (MA-ESO)..................1

1.2 Calculation of the yield of acetoacetylated soybean oil (MA-ESO-TBA).......2

2. The GPC of acetoacetylated soybean oil...2

3. Model reaction...2

4. The DSC curves indicating glass transition of the four films (P1, P2, P3, P4) 3

1. Calculation of the yield of acetoacetylated soybean oil by ^{1}H NMR

1.1. Calculation of the yield of soybean oil-based polyols (MA-ESO)

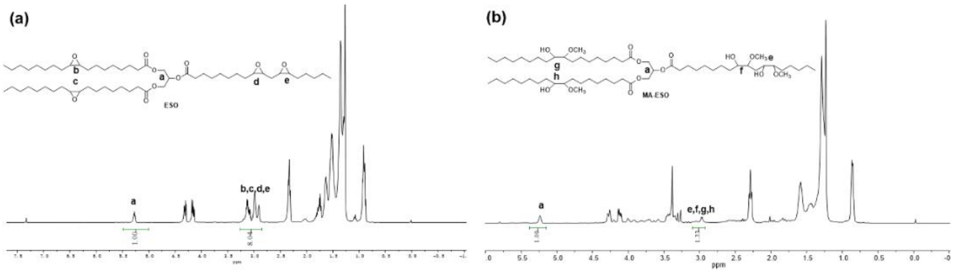

Figure S1. ^{1}H NMR spectra of (a) epoxy soybean oil (ESO) and (b) soybean oil-based polyols (MA-ESO).

Figure S1a shows that the peak area of a is 1, and the peak areas of b, c, d and e are 8; thus, the ^{1}H NMR peak areas correspond to the compound structure. Therefore, we can calculate the yield on the basis of the peak area ratios. The yield of soybean oil-based polyols (MA-ESO) W (%) = [A(b + c + d + e)–A(e + f + g + h)]/A(b + c + d + e) = (8-1.33)/8 = 84%.

1.2. Calculation of the yield of acetoacetylated soybean oil (MA-ESO-TBA)

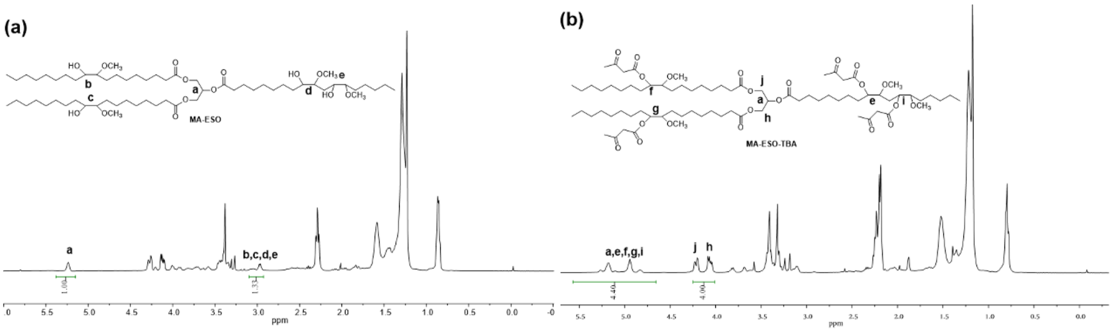

Figure S2. ^{1}H NMR spectra of (a) soybean oil-based polyols (MA-ESO) and (b) acetoacetylated soybean oil (MA-ESO-TBA).

The calculation method is the same as in 1.1, and the yield of modified soybean oil (MA-ESO-TBA) W (%) = [A(a + f + e + i)–A(a)]/4 = (4.40-1)/4 = 0.85%

2. The GPC of acetoacetylated soybean oil

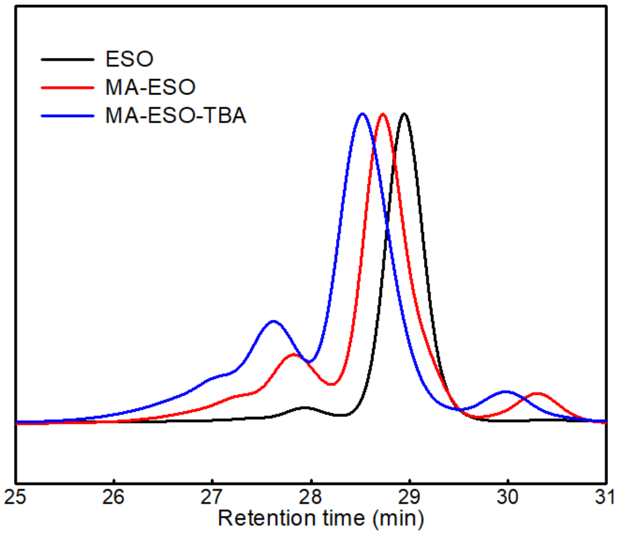

Figure S3. The GPC of acetoacetylated soybean oil.

Table S1. Properties of the acetoacetylated soybean oil.

Sample	Viscosity (Pa s^{-1}) at 25 °C	M_n	M_w	PDI
ESO	0.14	878	970	1.07
MA-ESO	1.31	1006	1057	1.05
MA-ESO-TBA	0.61	1169	1216	1.04

3. Model reaction

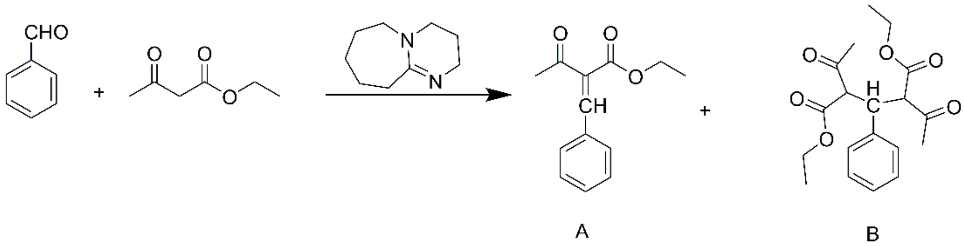

A 50 mL round bottomed flask equipped with a magnetic stir bar and a condenser was charged with ethyl acetate (2 g, 15.4 mmol, 1eq), benzaldehyde (1.22 g, 11.5 mmol, 0.75 eq) and 1,8-diazabicycloundec-7-ene (equal to 5 *wt* % of the total weight) in THF (10 mL). The reaction mixture was stirred at room temperature for 36 h. The solution was concentrated and the crude product was purified with column chromatography (CH_2Cl_2) to give a yellow oil of **compound A** (43% yield) and a white solid of **compound B** (41% yield). **Compound A**, ^{1}H NMR ($CDCl_3$, 400 MHz): δ(ppm) = 7.58 – 7.56 (t, 1H), 7.47 -7.44 (t, 2H), 7.39 -7.37 (t, 2H), 4.36 -4.30 (m, 4H), 4.20 – 4.17 (t, 1H), 3.45 - 3.43 (d, 2H), 2.42 (s, 6H), 1.29 - 1.25 (t, 6H). ^{13}C NMR ($CDCl_3$, 400 MHz): δ(ppm) = 194.15, 167.28, 140.77, 130.21, 129.03, 128.35, 127.56, 61.20, 60.85, 49.62, 26.02, 13.37.**Compound B**,^{1}H NMR ($CDCl_3$, 400 MHz): δ(ppm) = 7.31 - 7.29 (d, 2H), 7.27 - 7.25 (t, 2H), 7.23 - 7.21 (t, 1H), 4.05 - 3.98 (m, 2H), 3.87 – 3.83 (m, 2H), 3.73 – 3.67 (t, 1H), 3.05 - 3.03 (d, 1H), 2.74 – 2.71 (d, 1H), 1.63 (s, 3H), 1.35 (s, 3H), 1.06 – 1.02 (t, 3H), 0.83 - 0.78 (t, 3H). ^{13}C NMR ($CDCl_3$, 400 MHz): δ(ppm) = 202.63, 175.25, 169.07, 139.63, 130.08, 129.54, 129.21, 74.48, 63.99, 62.47, 58.52, 54.22, 46.72, 30.10, 15.38, 15.05.

4. The DSC curves indicating glass transition of the four films (P1, P2, P3, P4)

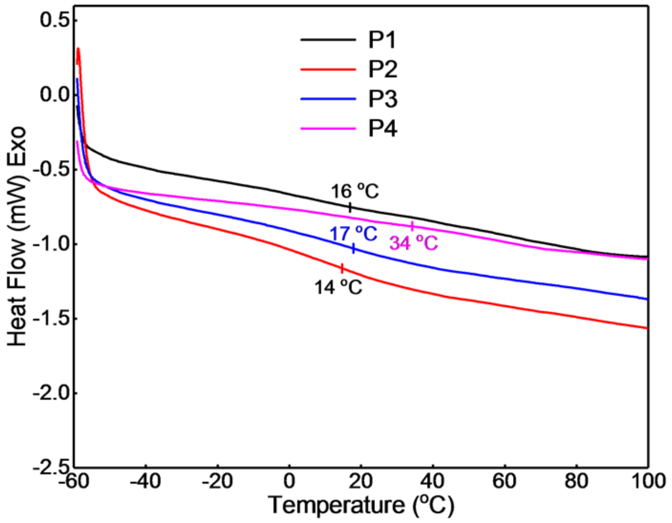

Figure S4. DSC curves indicate the glass transition temperature (T_g) of the four films.

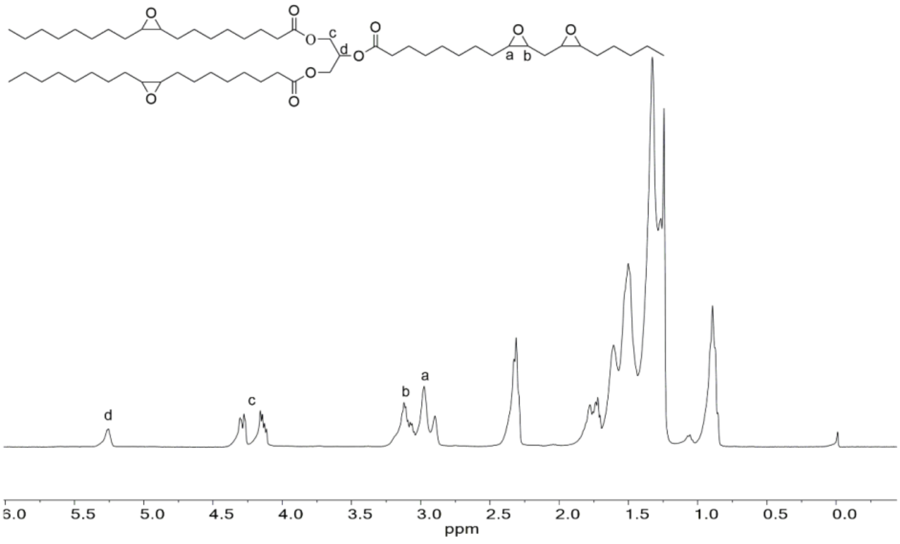

Figure S5. ^{1}H NMR (400 M) spectrum of epoxy soybean oil (ESO).

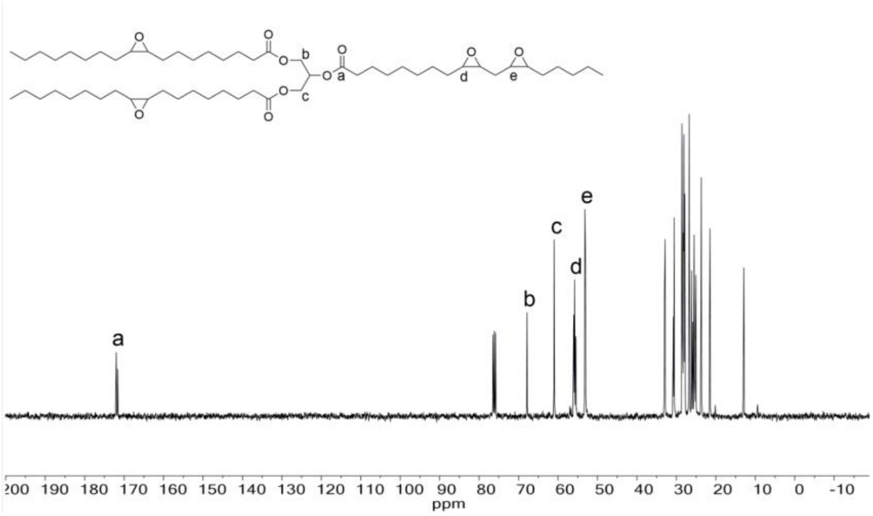

Figure S6. ^{13}C NMR (400 M) spectrum of epoxy soybean oil (ESO).

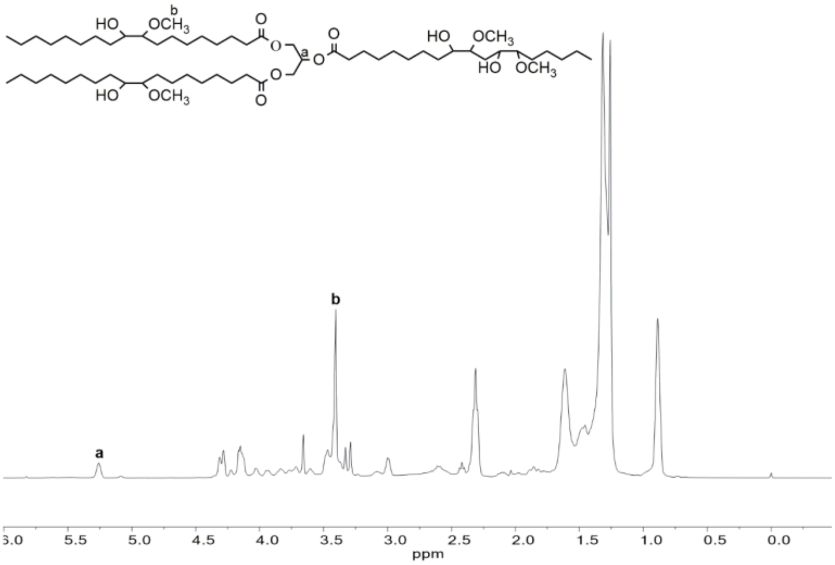

Figure S7. ^{1}H NMR (400 M) spectrum of soybean oil-based polyols (MA-ESO).

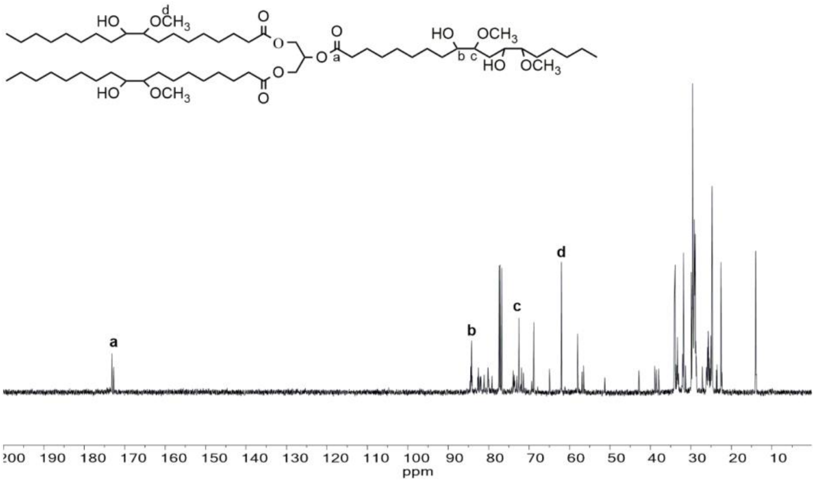

Figure S8. ^{13}C NMR (400 M) spectrum of soybean oil-based polyols (MA-ESO).

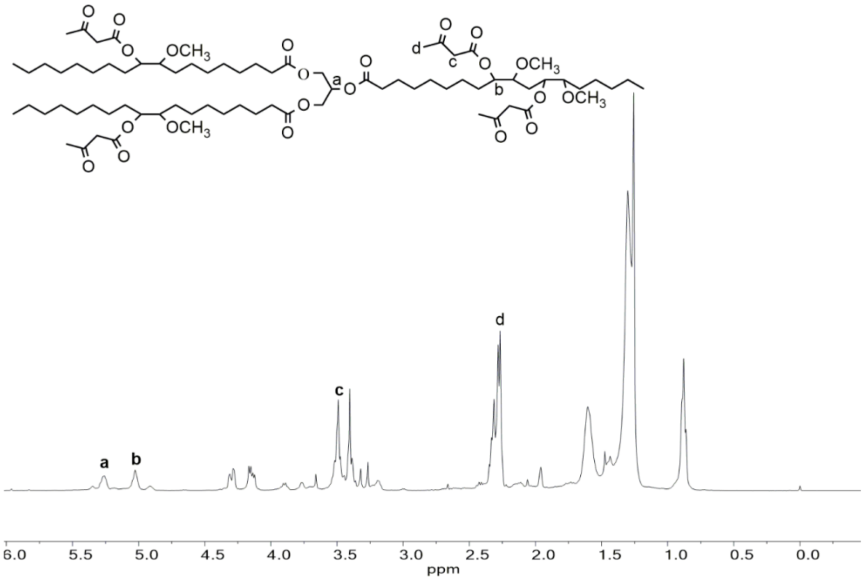

Figure S9. ^{1}H NMR (400 M) spectrum of acetoacetylated soybean oil (MA-ESO-TBA).

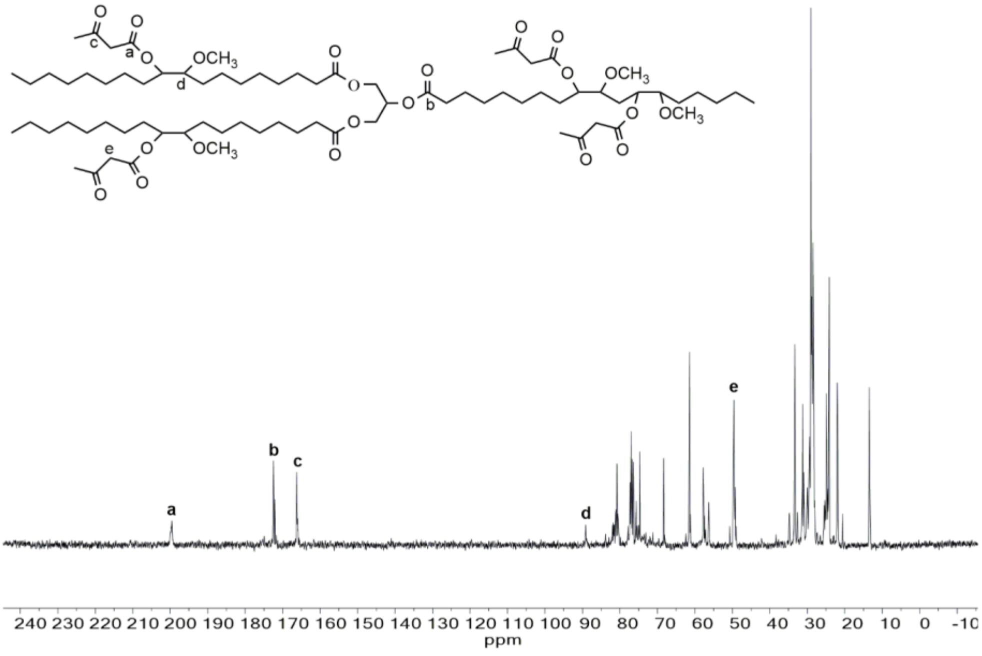

Figure S10. ^{13}C NMR (400 M) spectrum of acetoacetylated soybean oil (MA-ESO-TBA).

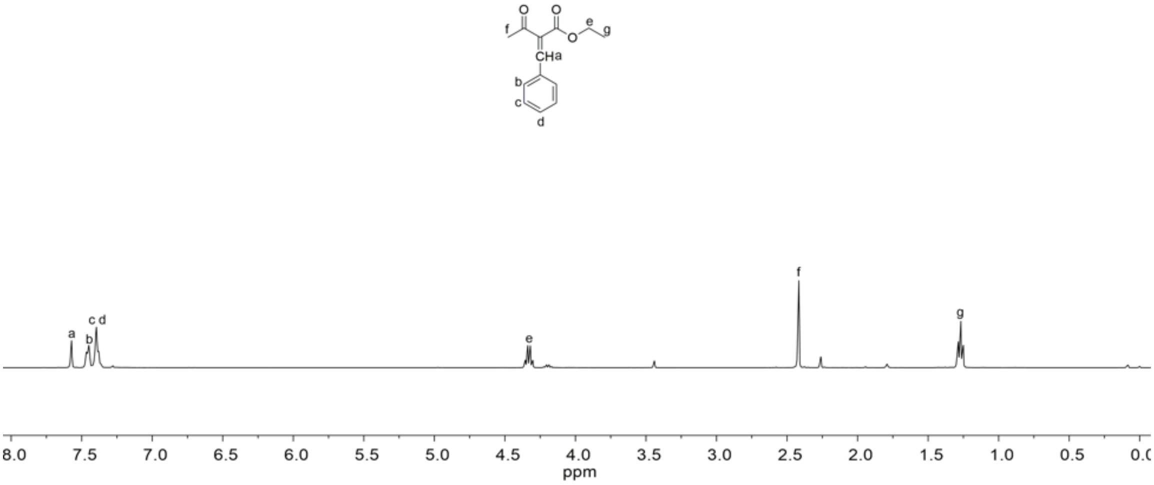

Figure S11. ^{1}H NMR (400 M) spectrum of **compound A.**

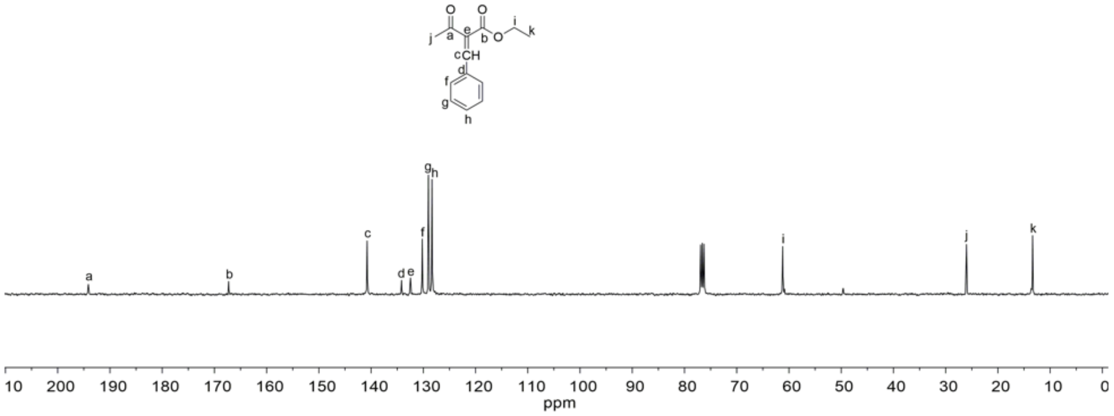

Figure S12. ^{13}C NMR (400 M) spectrum of **compound A.**

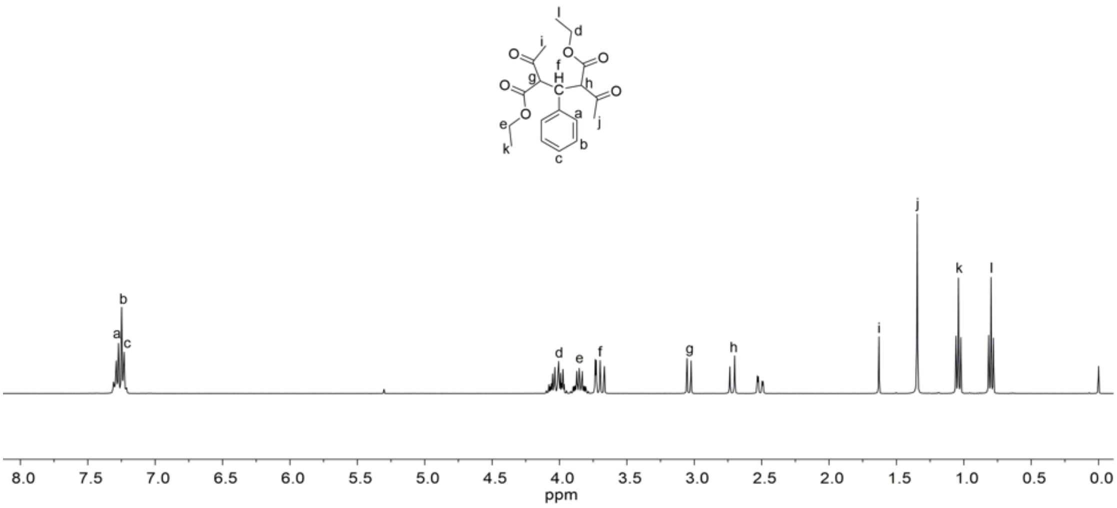

Figure S13. ^{1}H NMR (400 M) spectrum of **compound B.**

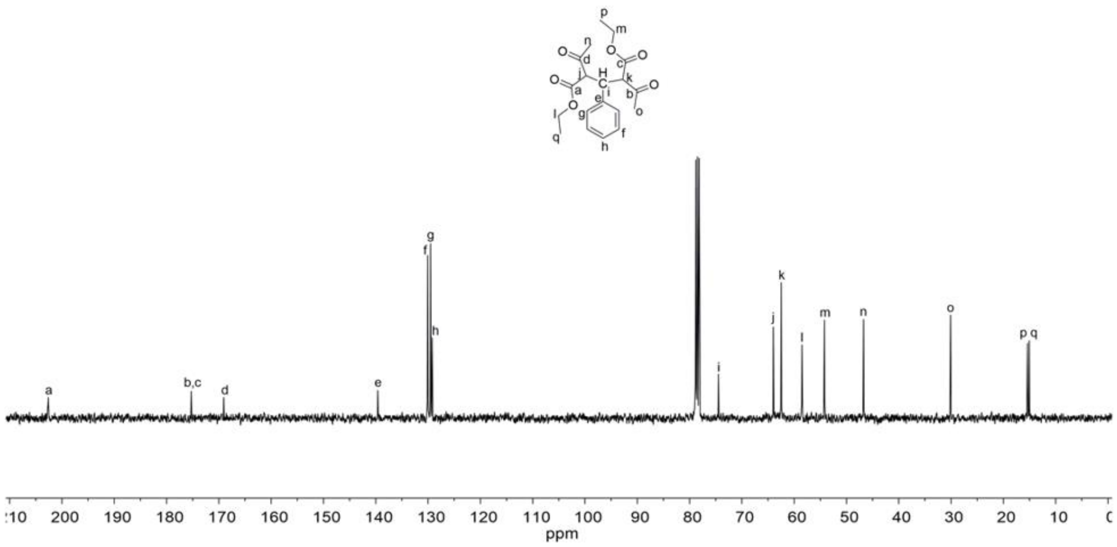

Figure S14. ^{13}C NMR (400 M) spectrum of **compound B.**
